# Anatomical Material

**Published:** 1881-05

**Authors:** 


### ANATOMICAL MATERIAL.

That the public are awakening to the fact that the study of practical anatomy is one of vital importance to those who are preparing themselves for the practice of medicine is evidenced by the interest which the legislators of some of our states are taking in framing laws to afford facilities and protection for those who wish to dissect, and in so doing protect their own lives. This movement is somewhat due to the flood of quacks practicing under cover of lying diplomas, with which the country is flooded, and Dr. Buchanan's college, was not the only one guilty of this sort of thing; for there are other institutions of more respectable pretentions doing this same thing with the only excuse "others do it, why not we?" Dr. B. & Co. deserve some credit for opening the eyes of the people and starting a reform. "Give the d—l his due."

The following is a copy of a law recently enacted by the legislature of Michigan:

"AN ACT

To amend sections 1 and 2 of act number 138 of session laws of 1875, relative to subjects for dissection for the advancement of science, approved April 27, 1875, the same being sections 2110 and 2111 of chapter 65 of the compiled laws of 1871, as amended.

"SEC. 1. *The People of the State of Michigan enact,* That sections 1 and 2 of act number 138 of the session laws of 1875, approved April 27, 1875, being sections 2110 and 2111 of chapter 65 of the compiled laws of 1871, as amended so as to read as follows:

"(2110). SEC. 1. Any member of either of the following boards, and any of the following named officers or persons, to-wit: The board of health of any city, village or township, the common council of any city, the board of trustees of any village, the mayor of any city, president of any village, any board, or officer having the direction, management, charge, or control, in whole, or in part, of any prison, house of correction, work-house, jail or lock-up, county superintendents of the poor, keepers of poorhouses and almshouses, any physician, or other person in charge of any poor-house or alms-house, sheriff, coroners, the board of State commissioners, the board of trustees, board of control, and all officers, physicians, and persons in charge, in whole or in part, of any institution for the deaf and dumb, blind, and insane, or other charitable institution founded or supported, in whole, or in part, at public expense, having in his or their possession or control the dead body of any person not claimed by any relative, or legal representative, as hereinafter provided, and which may be required to be buried at public expense, or the expense of any one of such public or charitable institutions, shall deliver such dead body or bodies, within thirty-six hours after death, or after he or they shall become possessed thereof, to the express or railway company at the nearest railway station, placed in a plain coffin and enclosed in a strong box, securely fastened, and plainly directed to the "Demonstrator of anatomy, of the University of Michigan," Ann Arbor, Michigan," excepting only the bodies of such persons as shall have died from some infectious disease. And such boards, common councils, officers, or other persons making such shipment shall take the usual shipping receipt for such package, and shall notify the consignee of such shipment by letter, mailed on the day the package is so delivered as aforesaid; and shall also inclose in such letter a statement, giving, as nearly as can be ascertained, the name, age, residence, and cause of death of such deceased person; and the name and post-office address of the known relatives of such deceased person, whose body has been shipped as aforesaid; and also a statement of the costs and expenses which have been incurred in the procuring of the coffin, box, preparation of body for shipment, and shipping the same. And, upon the receipt of such consignment, the said demonstrator of anatomy of the University of Michigan shall immediately forward to such officers, board, council, or institution, or persons making such shipment, or incurring such expenses, the amount thereof, not exceeding in any case the sum of fifteen dollars: *Provided*, Such dead body shall not be so shipped or delivered as aforesaid, if it shall be requested in good faith for interment by any relative before the same shall be shipped as aforesaid, and in case the dead body of any person, so delivered or shipped as aforesaid, be subsequently claimed or demanded of said demonstrator of anatomy, or of any other person or institution, into whose possession or under whose control it may have been placed, by virtue of the provisions of this law, by any relative or legal representative of such deceased person, for private interment, it shall be given up by such claimant even after the same shall have been interred, as hereinafter provided. Such bodies shall be used only for the purposes hereinafter mentioned, and shall then, in all cases, be interred in some suitable place, kept for that purpose, and a correct record shall be kept of every such body, and all matters by which such body may be identified coming to the knowledge of the person or officers at any time in charge of such bodies, shall be faithfully recorded at length in a book to be kept for such purposes, to the end that the same may be at any time traced and recovered by the friends and relatives of such deceased person: *And provided further*, That the institution, board, council, officer, or person aforesaid in charge of any such body as aforesaid shall, immediately after the death of such person, notify, if possible, by telegraph, or otherwise by letter, one or more of the nearest known relatives of such deceased person of the death of such person; and in no case shall the body of any such deceased person be delivered or shipped as aforesaid until after the expiration of twenty-four hours from death; and every individual officer or party violating any of the provisions of this section shall be deemed guilty of a misdemeanor.

"(2111). Sec. 2. The bodies so delivered, or shipped as aforesaid, shall be used for the advancement of anatomical science in this State and in the following institutions of learning only, viz.: The University of Michigan, Detroit Medical College, and Michigan College of Medicine. And said bodies shall be distributed to and among the same equitably, and in the order in which they are received and the number assigned to each by said demonstrator of anatomy, shall be proportional to that of its students in actual attendance. And each of said institutions shall pay quarterly to said demonstrator its ratable proportion of the expenses borne and incurred under this act: *Provided, however,*

That said demonstrator of anatomy, upon the receipt of every body, under and by virtue of the provisions of this act, shall cause the same to be embalmed or put in a state of preservation, and shall not permit the same to be delivered to either of said institutions for the purpose of dissection, until the same shall have been in his possession at least ten days. And it shall be the duty of said demonstrator of anatomy, upon the receipt of every body, to immediately notify the relatives of such deceased person, if known, of the receipt of such body, either by mail or telegraph, as he may deem best. And that said body will be preserved intact, for the space of ten days, in which time such relative will be entitled to said body for the purpose of private interment, upon payment of the expenses already incurred. And if the relatives or legal representative of such deceased person shall request said body for the purpose of interment, and shall pay said expenses, said demonstrator shall deliver to such relative or legal representative, the said body, together with the said coffin and box inclosing the same. But in case said body shall not be requested by such relatives until after the same shall have been applied to the purposes intended, the remains thereof, together with the coffin and box aforesaid, shall be delivered without charge: *Provided*, That the University of Michigan, Detroit Medical College, and Michigan College of Medicine aforesaid, and each and every other medical institution shall not receive into their possession any bodies procured in this State other than those provided for by the provisions of this act, and every individual or party violating this provision shall be deemed guilty of a misdemeanor.

"(2112). SEC. 3. No such dead body shall be sold or delivered to any person to be taken out of the State, nor shall any such dead body be shipped to any person or place out of the State, or be used within the State for any purpose except for the prosecution of anatomical science. Any person violating any of the provisions of this act shall be punished by a fine of not less than fifty or more than one hundred dollars, or by imprisonment in the county jail not less than one or more than three months, or by both such fine and imprisonment, at the discretion of the court

"This act is ordered to take immediate effect.

"Approved, March 2d, A. D. 1881.

